# Exploring the Relationship of Antioxidant Characteristics and Fatty Acids with Volatile Flavor Compounds (VOCs) by GC-IMS and GC-O-MS in Different Breeds of Pigs

**DOI:** 10.3390/foods14203580

**Published:** 2025-10-21

**Authors:** Xinyuan Huang, Hui Liu, Xiaoyan Tang, Yuhui Zhang, Yaxuan Li

**Affiliations:** 1 College of Food Science and Technology, Nanjing Agricultural University, Nanjing 210095, China; 2021808080@stu.njau.edu.cn (X.H.); suru6803@163.com (Y.L.); 2 Key Laboratory of Agro-Product Quality and Safety, Institute of Quality Standard & Testing Technology for Agro-Products, Chinese Academy of Agricultural Sciences, Beijing 100081, China; 2019208023@njau.edu.cn (H.L.); zyhtxdxx@163.com (Y.Z.)

**Keywords:** pork, antioxidant characteristics, fatty acids, volatile organic compounds (VOCs), GC-O-MS

## Abstract

The volatile organic compounds (VOCs) are the main flavor constituents of different pig breeds, which have positive effects on the quality evaluation of pork. This study aimed to clarify the effects of lipid oxidation on characteristic VOCs in different breeds of pigs. The fatty acid composition and antioxidant characteristics of the Ningxiang (NX) pig, Rongchang (RC) pig, Duroc × Wujin (DW) pig, and Duroc × Landrace × Yorkshire (DLY) pig were determined. The VOCs from these four pig breeds were analyzed by gas chromatography–ion migration spectrometry (GC-IMS) and solid-phase micro-extraction–gas chromatography–olfactory mass spectrometry (SPME-GC-O-MS). A total of 49 volatile compounds were identified by GC-IMS, whereas GC-O-MS detected 97 volatile components, including aldehydes, alcohols, ketones, acids, and esters. Among these, aldehydes and alcohols were the predominant categories. The results showed that RC breed pork had the highest fatty acid content, whereas NX breed pork exhibited the highest antioxidant activity. Among the VOCs from these four pig breeds, tridecanal showed a strong positive correlation with antioxidant capacity (T-AOC) and vitamin E, which was mainly reflected in NX. Furthermore, the key VOCs across the different pig breeds were mainly related to unsaturated fatty acids, such as C20:3n6, C18:1n9c, and C18:2n6c. In conclusion, the antioxidant characteristics of NX pigs are closely associated with their unique volatile flavor profile, while the characteristic flavor compounds across different pig breeds are primarily influenced by the composition and oxidation of unsaturated fatty acids.

## 1. Introduction

The taste of pork plays a crucial role in the quality of meat, and consumers’ focus on pork flavor is intensifying, making it a key determinant for selecting pork [1]. Although some varieties of lean meat on the market grow rapidly and have a high conversion rate, they have a poor meat taste [2]. Intramuscular fat in pork has been reported to have a positive effect on flavor quality [3,4,5]. The breed of pig is considered to be one of the important factors that directly affect the intramuscular fat content [6]. Compared with DLY (Duroc × Landrace × Yorkshire), regional breeds in China typically possess a greater amount of fat within muscles, attributed to their slower growth rate and extended growth duration. The flavor of fresh meat is minimal, and it has only a bloody flavor. However, cooked meat undergoes various chemical processes like lipolysis, lipid oxidation, streaker degradation, and the Maillard reaction, resulting in the creation of various metabolites, such as fatty acids and amino acids [7]. Further reactions of these metabolites produce a variety of flavor compounds, primarily aroma compounds [8]. Lipid oxidation and decomposition are key factors in producing pork’s volatile taste. The majority of volatile flavoring agents in cooked meats, such as aldehydes, ketones, and esters, originate from the breakdown of lipids into fatty acids and the thermal decomposition and oxidation of these acids [7]. Lipids are gradually hydrolyzed to free fatty acids under the action of endogenous enzymes (lipase, diglyceride lipase, lipoacylglycerol lipase, etc.) or heating [9]. A series of small-molecule aroma compounds such as ketones, aldehydes, alcohols, esters, furans, and lactones are produced by the oxidative decomposition of fatty acids through endogenous factors and exogenous factors. Aldehydes come from the oxidation of free fatty acids, alcohols, and ketones from the decarboxylation of β-ketoacids, and alcohols and carboxylic acids of intramuscular and intermuscular fats in meat can be esterified to form esters [10].

In recent years, with the increasingly mature research methods and technologies of volatile flavor substances, relevant reports have been increasing [11,12]. HS-GC-IMS has been widely used in food, medicine, environment, medical and other industries because of its advantages of simple operation, high detection sensitivity (ppb level), fast response (30 ms) and so on [11]. In addition, GC-MS analysis has the advantages of high separation efficiency, high sensitivity, simple quantitative and strong qualitative ability [12]. GC-MS is commonly used for qualitative and quantitative determination of volatile organic compounds [13]. Often, flavor studies of samples using different extraction methods and analytical test instruments can produce more comprehensive data.

Currently, both local and international research on pork primarily concentrates on the accelerating growth, detecting nutritional content, and processing techniques to improve pork quality [14,15,16], however, the related research into the properties of volatile flavors and the origins of variances between different breeds of pigs and regular lean pigs are scarce. In this study, the VOCs of Ningxiang pig (NX), Rongchang pig (RC), Duroc × Wujin pig (DW) and DLY were analyzed by GC-IMS and GC-O-MS, and the fatty acid and antioxidant properties of different breeds of pigs were compared to evaluate the influence of lipid oxidation process on the formation of flavor. This will deepen our understanding of the unique flavors of different breeds pigs and their sources, and provide a sound scientific basis for improving pork flavor.

## 2. Materials and Methods

### 2.1. Sample and Chemical Reagents

Samples of the longissimus dorsi muscle were collected from Ningxiang pigs (NX) at the Subtropical Agroecology Institute, Chinese Academy of Sciences, Hunan Province; Rongchang pigs (RC) at the Chongqing Academy of Animal Science; Duroc × Wujin pigs (DW) at the Laopu domesticated breeding base, Xuanwei, Yunnan Province; and DLY pigs. Each breed consisted of six animals. Pigs were slaughtered following electrical stunning and exsanguination at a local slaughterhouse, after the completion of the fattening period. All procedures adhered to the ‘Regulations on Administration of Hog Slaughter’ and ‘Good Manufacturing Practices for Pig Slaughter (GB/T 19479-2004 [17])’ in China. The left longissimus dorsi muscle from each pig was harvested, vacuum-packed, and stored at −20 °C until analysis.

The analytical purity of all chemicals was greater than 98%. The standard compound C4–C9 n-alkanes (Hanon Group, Jinan, China) were used as the calibration fluid for GC-IMS analysis. 2-methyl-3-heptanone (Sigma-Aldrich, Shanghai, China) was used as an internal standard for GC-MS. Additionally, C7–C40 n-alkanes (Sigma-Aldrich, Shanghai, China) were employed to determine the linear retention index (LRI).

### 2.2. Analysis of VOCs

#### 2.2.1. HS-GC-IMS Analysis in Different Varieties of Pork

The method used was adapted from Wu et al. [18] with modifications. Minced pork samples from four varieties were thawed at 4 °C for 12 h. A 3.00 g portion of each sample was weighed and placed in a 20 mL headspace bottle, which was then heated at 100 °C in a constant-temperature water bath for 15 min. Each sample was analyzed in triplicate. The samples were analyzed for volatile organic compounds (VOCs) using an SE-5 chromatographic column on a GC-IMS system. Prior to analysis, the system was calibrated using C4–C9 n-alkanes.

The samples were incubated at 60 °C with agitation at 500 rpm for 20 min before analysis. A 500 μL injection volume was used, with the GC column temperature set to 60 °C and IMS to 45 °C. The carrier and drift gases were high-purity nitrogen (purity ≥ 99.999%), with an initial flow rate of 2 mL/min, increasing to 10 mL/min for 0 to 10 min, and 100 mL/min for 10 to 25 min. The drift gas flow rate was maintained at 150 mL/min.

#### 2.2.2. SPME-GC-O-MS Analysis

This method was also adapted from Wu et al. [18] with modifications. A 50 μm DVB/CAR-PDMS SPME fiber (Supelco, Bellefonte, PA, USA) was aged at 240 °C for 40 min in the GC-MS injection port to minimize noise. Minced pork samples were thawed at 4 °C for 12 h, after which 3.00 g of each sample was weighed into a 20 mL headspace bottle, and 1 μL of 2-methyl-3-heptanone (0.816 μg/μL) was added as the internal standard. Samples were incubated at 100 °C for 15 min, followed by immersion in a 60 °C water bath for 20 min.

The SPME fiber was exposed to the headspace for 40 min to adsorb VOCs. After extraction, the fiber was transferred to the GC inlet for desorption at 250 °C for 5 min. The chromatographic columns used were a low-polar DB-5 (30 m × 0.25 mm × 0.25 μm) and a primary polar DB-WAX (30 m × 0.25 mm × 0.25 μm), both from Agilent Technologies (Shanghai, China).

GC-MS conditions followed those described by Wu et al. [18], with an oven temperature program of 35 °C for 3 min, increased to 130 °C at 4 °C/min, followed by a 2 min hold, then raised to 230 °C at 8 °C/min with an 8 min hold. Helium was used as the carrier gas at 2.0 mL/min, with splitless injection. The mass spectrometer operated in electron impact (EI) mode (ionization energy of 70 eV, source temperature 230 °C), acquiring mass spectra in full-scan mode (30–500 *m*/*z*). Sniffing conditions were as follows: the pipeline was maintained at 250 °C, with nitrogen flowing at 30 mL/min to prevent mucosal drying. Trained experimenters performed the sniffing analysis.

The VOCs were analyzed based on the retention index (RI) from n-alkanes C7–C40 and identified using the NIST database and spectral library. Quantification was based on peak areas using the internal standard (2-methyl-3-heptanone). Odor thresholds and flavor characteristics were referenced from the literature and online databases (https://pubchem.ncbi.nlm.nih.gov/; http://www.thegoodscentscompany.com/, accessed on 21 March 2024).

### 2.3. Analysis of Intramuscular Fat

The four varieties of pork mince were thawed at 4 °C for 12 h. The intramuscular fat content was determined using the solvent extraction method from GB 5009.6-2016 [19] “Determination of Fat in Food under National Standards for Food Safety”. Samples were dried at 105 °C for 8 h to remove moisture and then ground into powder. The fat was extracted using petroleum ether, and the fat content was calculated by the difference in weight after drying.

### 2.4. Analysis of Fatty Acids

The fatty acid content was measured using the external standard method described in GB 5009.168-2016 [20] “National Food Safety Standard—Determination of Fatty Acids in Foods”. Freeze-dried pork was powdered, and 0.500 g of meat powder was subjected to sequential extraction with toluene and 10% acetyl chloride in methanol at 80 °C for 2 h. Subsequently, taking a 50 mL centrifuge tube, the reaction solution was transferred and washed with 6% sodium carbonate solution. After washing, the solution was centrifuged, and the supernatant was analyzed by gas chromatography using mixed fatty acid methyl esters as external standards.

### 2.5. Analysis of Antioxidant Index

#### 2.5.1. Antioxidant Capacity Characterization Value Analysis

In this study, the antioxidant capacity of the pork samples was evaluated using multiple assays: total antioxidant capacity (T-AOC) based on the ferric reducing ability of plasma (FRAP) method and ABTS+ radical scavenging assay. Additionally, DPPH and hydroxyl radical inhibition (OH•−) were measured. The respective assay kits (Nanjing Jiancheng Bio-technology Co., Ltd., Nanjing, China) were used according to the manufacturer’s instructions.

#### 2.5.2. Endogenous Antioxidant Factor Analysis

Endogenous antioxidant factors, including glutathione peroxidase (GSH-Px), superoxide dismutase (SOD), catalase (CAT), total phenol content (TPC), vitamin C, and vitamin E, were determined. Enzyme activity was measured using commercial enzyme assay kits (Nanjing Jiancheng Biotechnology Co., Ltd.).

##### Extraction and Activity Determination of Antioxidant Enzymes

Enzyme activity was determined following the manufacturer’s instructions for the GSH-Px, SOD, and CAT kits (Nanjing Jiancheng Biotechnology Co., Ltd.).

##### Total Phenol Content (TPC) Analysis

TPC was measured following the method of Wootton Beard et al. [21], with slight modifications. Pork samples were homogenized, and the supernatant was treated with Folin–Ciocalteu reagent and sodium carbonate solution. The absorbance at 760 nm was measured using a Tecan Infinite 200 PRO UV–vis spectrophotometer (Tecan, Switzerland), and TPC was expressed as milligrams of gallic acid equivalents (GAEs) per gram of muscle.

##### Determination of Vitamin C and Vitamin E Content

Vitamin C and vitamin E content were determined using the corresponding assay kits (Nanjing Jiancheng Biotechnology Co., Ltd., Nanjing, China, A008-1-1) according to the manufacturer’s guidelines.

### 2.6. Data Analysis

The volatile organic compound data were analyzed by the Reporter plug-in and Gallery Plot plug-in of GC-IMS to compare the volatile organic compound fingerprints of pork from different breeds. Qualitative analysis was performed using instrument-configured NIST and IMS databases.

Relative odor activity value (ROAV): ROAV is proposed to explain the contribution of the relative concentration of individual volatile compounds to the overall aroma [22,23]. ROAV is used to identify key volatile compounds in different breeds of pigs, with a range of 0 to 100. The ROAV calculation method is based on the ratio of the odor activity value (OAV) to the highest odor activity value of the volatile components. The odor threshold of each volatile component was obtained by referring to the relevant literature.

Data were expressed as means ± standard error (SE), and statistical significance was set at *p* < 0.05. Statistical analysis was conducted using SPSS (version 22) and SIMCA 14.1 (Umetrics, Sweden) for PLS-DA.

## 3. Results and Discussion

### 3.1. Analysis of VOCs in Different Varieties of Pork

#### 3.1.1. VOCs Identified by GC-IMS in Different Varieties of Pork

**Figure 1:**
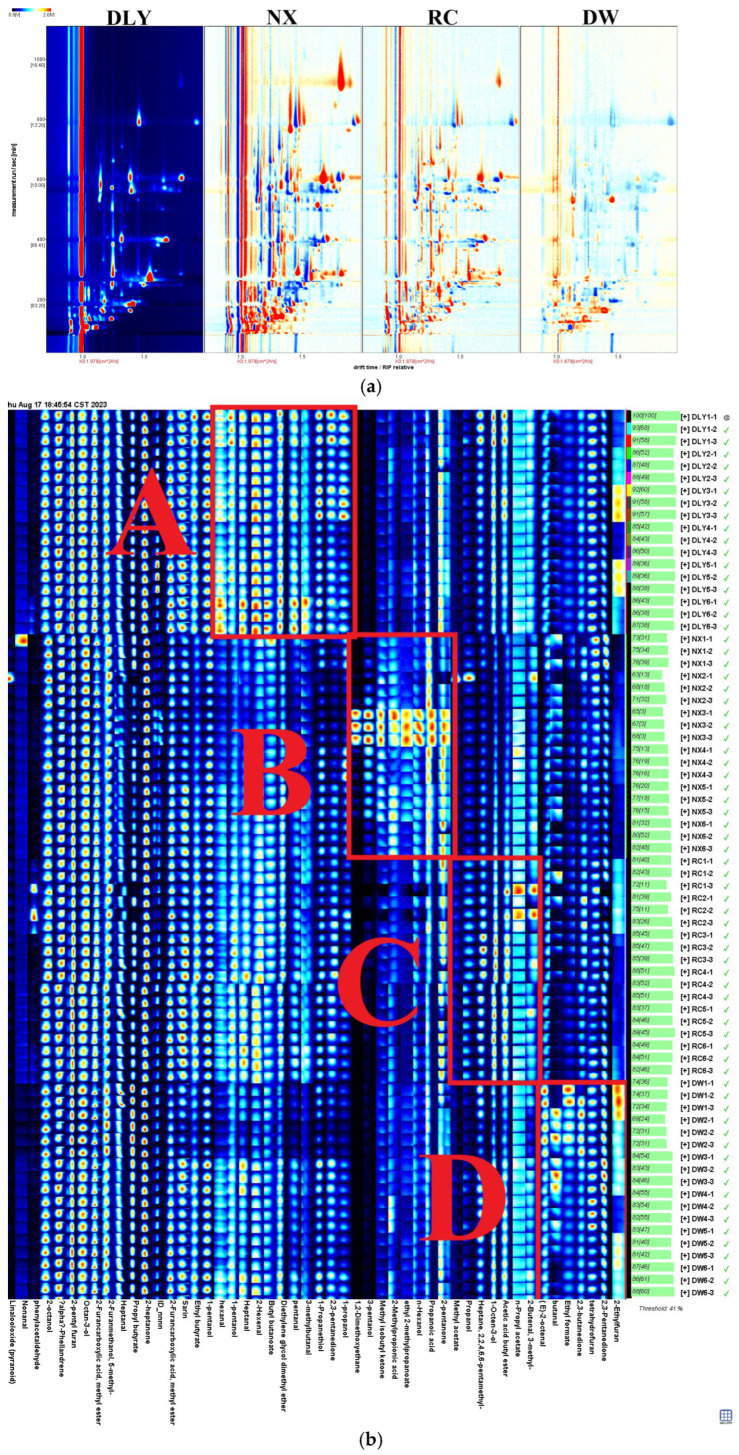
(**a**) Comparison of differences in 2D GC-IMS spectra of VOCs in pork from different breeds; (**b**) fingerprints of VOCs isolated from pork from different breeds using GC–IMS. DLY: Duroc × Landrace × Yorkshire; NX: Ningxiang pig; RC: Rongchang pig; DW: Duroc × Wujin.

Following the HS-GC-IMS analysis of VOCs for DLY, NX, RC, and DW in four pork varieties (Supplementary Material Table S1), consistency in VOCs among these pork types results in a white background post-deduction, red signifying a substance concentration exceeding the reference, and blue denoting a concentration below the reference [24]. As depicted in Figure 1a, the total aroma compounds levels in NX and RC exceed those in DLY, whereas DW’s levels fall below those in DLY. For the purpose of creating VOC profiles in DLY, NX, RC, and DW pork types, and to examine the variances in VOCs across these pork types, the VOCs for each pork type were ascertained using the NIST library and IMS database, in conjunction with the retention index (RI), retention duration, and drift period. Results from the qualitative analysis are presented in Schedule 1, with an initial identification of 50 VOCs, comprising 12 aldehydes, 11 alcohols, 11 esters, 6 ketones, 4 hydrocarbons, 2 acids, 3 furans, and 1 ether compound. As depicted in Figure 1b, the Gallery Plot add-on is capable of creating unique fingerprints by analyzing the signal values of every volatile substance in the GC-IMS spectrum, as evidenced by four pork samples. The findings indicated that while the flavor elements of the four pork types were alike, the overall composition of different substances varied. Regions A, B, C, and D stand out as the key elements of DLY, NX, RC, and DW. The A region encompasses nonanal, 2-octanol, n-hexanol, heptane, 2-heptanone, and 2-hexenal. The B region includes 2,3-butanedione, 2-ethylfuran, propanoic acid, ethyl formate, and 1-propanethiol. The C region includes heptanal(M), 2-methylpropionic acid, (E)-2-octenal, and 2-furanmethanol. The D region includes methyl isobutyl ketone, 3-methylbutanal, 2-pentanone, diethylene glycol dimethyl ether, 2-furancarboxylic acid, and butanal, etc.

**Figure 2:**
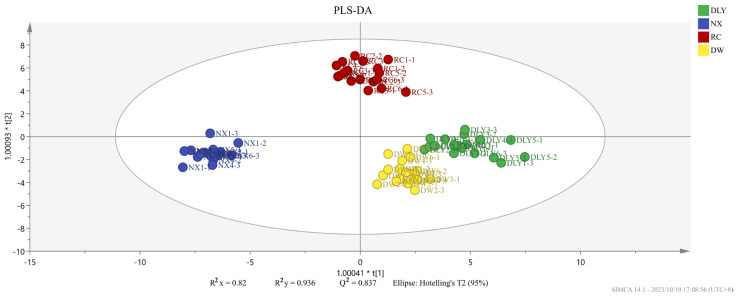
PLS-DA score plot of different varieties of pork.

To compare the recognition ability of HS-GC-IMS on VOCs across four pork varieties, we performed PLS-DA using the peak areas of 50 shared aroma compounds as the dependent variable and the pork types as the independent variable, and the resulting score plot clearly delineated the groups, as shown in Figure 2. The findings indicated variations in the volatile taste compounds across the quartet of pork specimens. The analysis result showed that the independent variable fitting index (R^2^x) stood at 0.891, the dependent variable fitting index (R^2^y) at 0.957, and the model prediction index (Q^2^) at 0.888, signifying satisfactory model fitting outcomes [25].

#### 3.1.2. VOCs Identified by GC-O-MS

In order to further understand the source of volatile flavor differences and study the characteristic aroma of different pork varieties, GC-O-MS with two columns of DB-WAX and DB-5 with different polarity were used to analyze and identify the aroma compounds and their relative contents. A total of 97 different aromatic elements underwent qualitative analysis, encompassing 25 aldehydes, 24 alcohols, 12 ketones, 8 acids, 7 esters, 9 hydrocarbons, 6 heterocyclic compounds, 3 phenols, 2 ethers, and 1 nitrogen-containing compound, among which 50 were common components. The percentage of aroma components in different varieties of pork and the difference analysis results are shown in Schedule 2. Among them, aldehydes were the largest, and their relative contents were consistent with the results of GC-IMS, accounting for up to 60% of all flavor substances, followed by alcohols and ketones. As one of the characteristic flavor products of fat degradation, aldehydes have a low threshold and complex taste, so their contribution to the overall flavor of meat is more prominent. Among them, saturated straight-chain aldehydes generally have a strong oily taste, which is a major characteristic flavor substance in fresh meat [26]. Caproaldehyde was the highest aldehyde detected in the samples of the four varieties of pork. Caproaldehyde is the oxidation product of linoleic acid and arachidonic acid [27], which can be used as an index to evaluate the oxidation stability and flavor acceptability of muscle meat [28]. In addition to aldehydes, the content of alcohols is also high, and the relative content in the four varieties of pork can reach 20%. Alcohols are mainly produced by the oxidative decomposition of lipids [29]. 1-octene-3-ol, with a strong mushroom flavor, is the alcohol substance with the highest content in the four varieties of pork, which comes from the degradation and oxidation of linoleic acid, and it has an important contribution to meat flavor [30]. Among the four varieties, the relative content of aldehyde and alcohol in DLY is the highest, which may be because the degree of oxidation of DLY is greater and the antioxidant activity of DLY is lower than that of the other three varieties of pork. In addition, there are some studies indicating that lipid oxidation is one of the pathways leading to the formation of volatile compounds, and high antioxidant activity can inhibit lipid oxidation [31,32]. In addition to aldehydes and alcohols, the content of 2, 3-octanedione and heterocyclic compounds with a fatty taste is high in the four varieties of pork.

**Figure 3:**
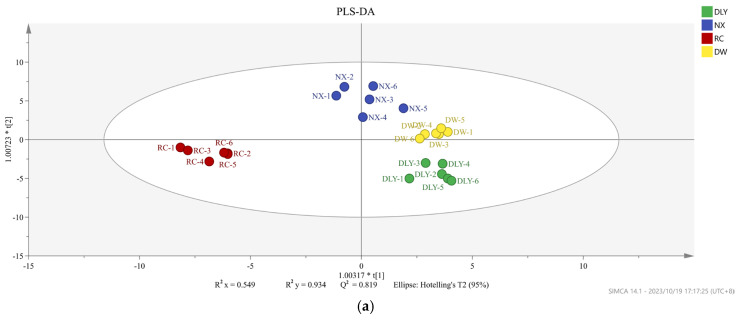

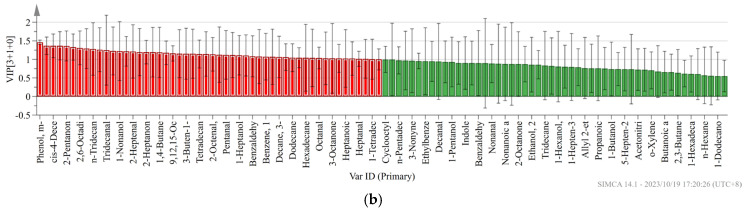
(**a**) PLS-DA scores of different pork varieties; (**b**) variable importance (VIP) factor values.

Not all volatile organic compounds in the sample have an effect on the flavor differences between different pork varieties. Based on the conditions of VIP value > 1 and *p* < 0.05, the PLS-DA model was constructed to distinguish the differences between pork varieties. We obtained an accurate model with a good fitting parameter (R2x = 0.549, R2y = 0.934, R2Y = 0.549, R2Y = 0.934, and Q2 = 0.819), and Figure 3 was drawn according to the analysis results. In the PLS-DA model, cis-4-decenal, 3-methyl-tetradecane, 2-heptanone, pentadecanal, tridecanal, hexanoic acid, 1-nonanol, (E)-2-decenal, and 48 other volatile compounds were VIP > 1.

The content of aroma components cannot be used to determine the aroma characteristics of pork. The ROAV value method is widely used to analyze key flavor compounds. When 0.1 ≤ ROAV < 1, there are modification effects on the overall odor modification components, and compounds with ROAV ≥ 1 are key active substances [26]. The threshold value of 1-octen-3-ol was 1 μg/kg, which was higher in the four kinds of pork and contributed the most to the overall flavor of the sample, so it was chosen as the benchmark for calculating the ROAV value in the four kinds of pork. The higher the ROAV value, the greater the contribution to pork aroma characteristics. Among the 48 key volatile compounds (VIP > 1, *p* < 0.05), a total of 11 key volatile compounds with ROAV value > 1 were identified (Table 1), including tridecanal (ROAV: 30.38), cis-4-decenal (ROAV: 11.32–20.33), 3-phenyl-2-propenal (ROAV: 7.17–14.71), (Z,Z,Z)-9,12, 15-octadecatrien-1-OL (ROAV: 9.37–11.51), etc. Tridecanal ROAV of NX was the highest, and it was detected only in NX. The smell was fresh clean petal grapefruit, which could give NX a unique fruit flavor. The 3-phenyl-2-propena ROAV is the highest in RC, which can be endowed with a strong cinnamon odor and is the main contribution to the aroma characteristics of RC. In both DW and DLY samples, cis-4-decenal has the highest ROAV, which has a fatty odor, which can in turn give DW and DLY a fatty aroma.

### 3.2. Analysis of Intramuscular Fat and Fatty Acid Spectrometry in Different Pork Varieties

Lipids mainly act as solvents and as the initial elements in making flavors for meat and related products [33]. Typically, flavor compounds are predominantly dissolved in lipids, which often experience lipolysis and oxidation to form flavor compounds [33]. Following lipid extraction, there was a notable decrease in the levels of fatty aldehyde and fatty alcohol in the volatile flavor of meat [34]. Flavor substances are primarily produced via flavor precursors’ lipid oxidation, the Maillard reaction, and the breakdown of thiamine [35]. The final products from the oxidation of lipids, such as aldehydes, alcohols, ketones, furans, and others, have significant volatility and a minimal olfactory threshold, which is crucial in shaping the distinct flavors of diverse meat items [8]. Alterations in the makeup of fatty acids may additionally influence the taste and quality of meat [36]. It is commonly believed that the self-oxidation of polyunsaturated fatty acids results in a foul smell, and the oxidation of proteins, peptides, and amino acids also can compromise their texture, flavor, and nutritional value [37]. To explore deeper into the link between flavor variances, flavor forerunners, and the lipid oxidation in four types of pork, the IMF and fatty acid levels in these pork varieties were ascertained. As shown in Table 2, the IMF content in RC was the highest, the IMF content in DLY was the lowest, and the IMF content in NX and DW was significantly higher than that in DLY (*p* < 0.05). This result is consistent with previous studies on IMF content in local breeds and commercial pigs [24]. The distribution patterns of fatty acids varied significantly across different pig breeds (*p* < 0.05), with NX showing maximal saturated fatty acid (SFA) proportions, DW attaining the highest monounsaturated fatty acid (MUFA) percentage, and RC achieving peak values for both unsaturated (UFA) and polyunsaturated fatty acid (PUFA) content. Previous studies have found that the main saturated fatty acids (SFAs) in pork are C18:0 and C16:0 [38], and the main MUFA is C18:1n9c. Our results show that the MUFA with the highest content in the four pork varieties analyzed is C18:1n9c, followed by C16:0, C18:0, and C18:2n6c. C18:2n6c is degraded by lipoxygenase to trans-11-octadecadienoic acid and then oxidized to 1-octene-3-ol, with a strong mushroom flavor, which is thought to contribute to the meat flavor [30]. Some researchers have found that the breed of pig significantly affects the composition of fatty acids in muscular tissues, and the flesh of pigs that grow at a slower pace often has a more favorable fatty acid profile, showing a higher proportion of PUFAs [39]. In this experiment, the PUFA ratio of RC was significantly higher than that of the other three varieties of pork. In addition, high levels of oleic acid and a high MUFA/PUFA ratio can promote the pleasing flavor of processed meat products [36]. The proportion of saturated fatty acids (SFAs) in NX is significantly higher than the other three pork varieties, and the ratio of unsaturated fatty acids (UFAs) is markedly lower than RC pork types, suggesting NX is less susceptible to lipid oxidation than the remaining pork types, or the extent of lipid oxidation is less than that of the other three. In addition, since the IMF content of DLY was significantly lower than that of the other three pork varieties, the absolute content of each fatty acid in DLY was lower than that of the other three kinds of pigs. Previous studies have also found that as IMF levels in pork increase, both SFAs and MUFAs also increase [40].

### 3.3. Analysis of Antioxidant Capacity Characterization Value and Endogenous Antioxidant Factors

The measurement results are presented in Table 3. NX exhibited the highest FRAP and ABTS•+ radical scavenging activities, whereas DLY showed the lowest FRAP. No significant differences in ABTS•+ scavenging capacity were observed among RC, DW, and DLY. These variations may be attributed to differences in the types and levels of antioxidant compounds present in different pork varieties. Wei Chen et al. [41] reported that Chinese native and hybrid pig breeds generally display higher total antioxidant capacity than DLY. The results of DPPH free radical scavenging ability measurement of four local varieties of pork are shown in Table 3; the DPPH free radical scavenging activity was highest in NX, significantly greater than that of RC (*p* < 0.05). In contrast, DW and DLY showed the lowest DPPH scavenging activity, both significantly lower than RC (*p* < 0.05). Hydroxyl radicals (OH•−), being the most reactive species among ROS, play a critical role in the oxidation of proteins, lipids, and nucleic acids. Results of OH•− inhibition revealed that DW exhibited the strongest hydroxyl radical scavenging ability, significantly higher than NX, RC, and DLY (*p* < 0.05), while no significant differences were found among the latter three breeds (Table 3).

The activities of endogenous antioxidant enzymes are also summarized in Table 3. Among the four breeds, NX showed the highest SOD and GSH-Px activities, whereas DW exhibited the greatest CAT activity. DLY had the lowest SOD and CAT activities, while the GSH-Px activity in DW was the lowest, but not significantly different from that of DLY. These differences in enzyme activities may partly explain the variation in antioxidant capacity among breeds. Previous research has shown that CAT, SOD, and GSH-Px effectively delay lipid oxidation in beef [42].

Polyphenols exert antioxidant effects by scavenging free radicals, inhibiting radical-producing enzymes, and chelating metal ions [43]. However, reports on phenolic content in pork are limited. As shown in Table 3, RC had the lowest total phenol content, although the difference from NX and DW was not significant. DLY showed the highest phenolic content, which did not differ significantly from NX or DW.

Vitamins C and E function as water-soluble and lipid-soluble chain-breaking antioxidants, respectively, and protect lipids, proteins, and membranes from oxidative damage. As shown in Table 3, DW had the highest vitamin C content, significantly greater than RC and DLY, but not significantly different from NX. The highest vitamin E content was observed in NX, significantly higher than DLY, whereas RC and DW exhibited the lowest levels, both significantly lower than DLY (*p* < 0.05). Descalzo et al. [44] found that vitamin E in animal tissues affects the stability of lipids during the storage of meat.

### 3.4. Relationship of Antioxidant Factors and Fatty Acids with VOCs

The PLSR model is a multivariate data analysis technique that combines multiple linear regression, principal component analysis, and correlation analysis to effectively address the issue of multicollinearity among independent variables. It can be used to predict the sensory quality of new samples by using gas-chromatographic data of volatiles and is the most widely used statistical tool when correlating sensory and instrumental data to creating predictive models [45]. Typically, the speed and degree of fat burning in meat are contingent on the makeup and levels of fatty acids. With the rising levels of free fatty acids, lipids are further oxidized to produce many different volatile compounds [46]. This research identified the distinct volatile flavor substances of different breeds of pigs, and differences in volatile flavor substances were found under the same conditions (Supplementary Material Table S2). Subsequently, a comparison was made between the fat and fatty acid levels and antioxidant factors of different breeds of pigs, revealing distinct differences. To delve deeper into how lipid oxidation impacts specific flavor characteristics in different breeds of pigs, our research identified variances in endogenous antioxidant abilities and factors among the four breeds of pigs. We found that the creation of characteristic aroma substances of the four breeds of pigs is not only affected by the content of fat and fatty acids but also affected by its own antioxidant capacity. It may be due to the strong endogenous antioxidant capacity of NX and the low content of oxidation products. Chen et al. [41] also confirmed that meat from local pig breeds in China had a higher total antioxidant capacity.

**Figure 4:**
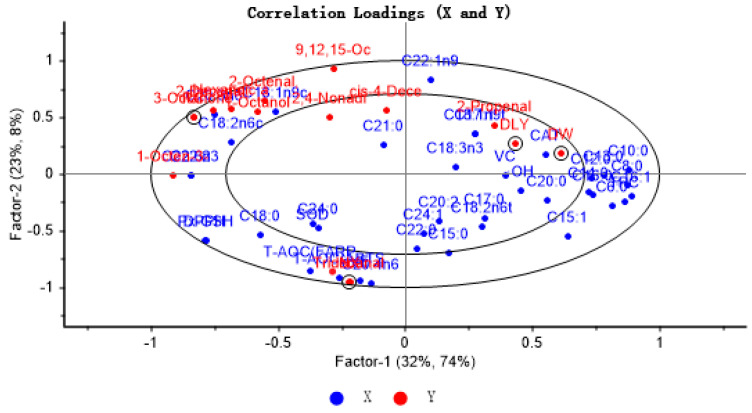
Correlation loading plot for VOCs with FFAs and lipid oxidation in four varieties of pork.

To explore the extent of association between key volatile components and antioxidant indices and fatty acids in four pork samples, the X matrix was labeled as antioxidant indices and fatty acids, while the Y matrix was identified as key volatile flavor compounds and four pork samples. PLSR provides a two-factor model that explains 55% of the variance of X (antioxidant markers and fatty acids) and 82% of the variance of Y (key VOCs and different pork varieties), with the inner and outer ellipses representing 50% and 100% of the variance of interpretation, respectively, with all variables in the ellipse. The results showed that the PLSR model could explain the relationship between antioxidant indices, fatty acids, and key flavor compounds in different breeds of pigs. As antioxidant indices and fatty acids approach the typical volatile aroma substances, the impact of these indices and fatty acids on the distinct volatile flavor compounds increases. As can be seen from Figure 4, the samples of RC and NX are located in the left outer ellipse of the figure, while the samples of DLY and DW pigs are located in the right inner ellipse. Most of the characteristic volatile flavor compounds were located between the two ovals and showed correlation with some antioxidant factors and unsaturated fatty acids. Among them, tridecyl aldehyde showed a strong positive correlation with antioxidant capacity (T-AOC) and vitamin E, which was mainly reflected in NX pig samples; this may be because NX pigs showed the highest vitamin E content and the strongest antioxidant capacity, while tridecyl aldehyde was only detected in NX pig samples. In addition to total antioxidant capacity and vitamin E, the other antioxidant markers were far away from the key flavor compounds, suggesting that other antioxidants had little effect on the key flavor compounds.

**Figure 5:**
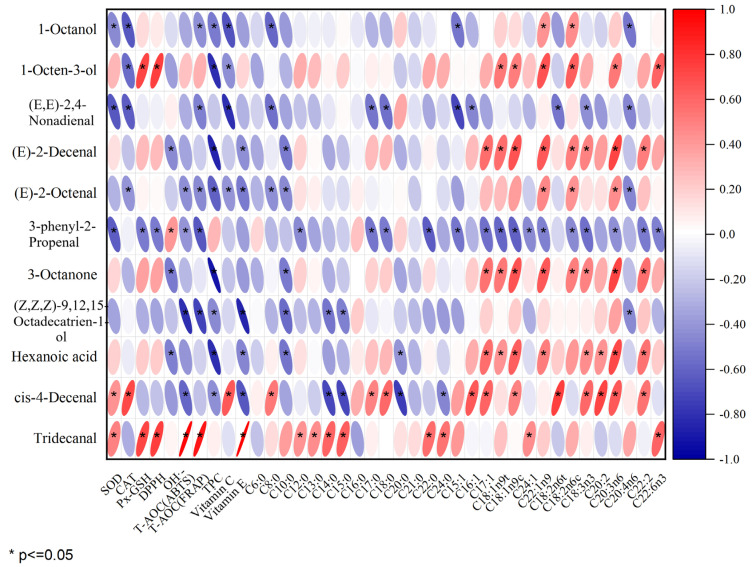
Pearson correlation analysis of key differentiating flavor compounds with fatty acids and antioxidant factors in four varieties of pork.

Figure 5 displays the outcomes of the Pearson correlation analysis, revealing a significant positive correlation of SOD with cis-4-decenal and tridecanal; CAT and total phenol were significantly positively correlated with cis-4-decenal; GSH-Px and DPPH were significantly positively correlated with 1-octen-3-ol and tridecanal. There was a notable positive association between OH•− and tridecanal, as well as T-AOC (ABTS), T-AOC (FRAP), and vitamin E with tridecanal. There was a notable positive association between C8:0, C17:0, and C18:0 with cis-4-decenal, while C12:0, C13:0, C15:0, C20:0, and C24:0 showed a significant positive correlation with tridecanal. The presence of C16:1 in unsaturated fatty acids showed a positive association with cis-4-decenal. There was a positive association between C17:1 and the compounds (E)-2-decenal, 3-octanone, hexanoic acid, and cis-4-decenal. There was a notable positive association between C18:ln9t and the compounds 1-octen-3-ol, (E)-2-decenal, 3-octanone, and hexanoic acid. C24:1 was positively correlated with tridecanal. C22:ln9t was significantly positively correlated with 1-octanol, 1-octen-3-ol, (E)-2-decenal, (E)-2-octenal, 3-octanone, and hexanoic acid; C18:2n6t was positively correlated with cis-4-decenal. C18:3n3 was positively correlated with (E)-2-decenal, 3-octanone, hexanoic acid, and cis-4-decenal. C20:2 was positively correlated with hexanoic acid and cis-4-decenal. Furthermore, C20:3n6, C18:2n6c C18:1n9c, C18:2n6c, C22:2, and C22:6n3 are positively correlated with 3-octanone. Notable links were found between essential volatile compounds like (E)-2-decenal, (E)-2-octenal, hexanoic acid, and 1-octen-3-ol. The association between (E)-2-decenal, (E)-2-octenal, hexanoic acid, and various other substances was greater than that of other pork varieties, as can be seen from Figure 5. These primary production of these compounds involved the oxidation of unsaturated fatty acids such as C20:3n6, C18:1n9c, and C18:2n6c. This may be due to the relatively high content of these unsaturated fatty acids in RC. In summary, there was a positive association between antioxidant factors and characteristic aroma compounds; a majority of unsaturated fatty acids were positively correlated with characteristic aroma compounds, and most saturated fatty acids were not significantly correlated with aroma compounds. Although a high correlation between volatile components and fatty acids may not imply causation, it might imply uniform changes in these variables [45]. As an illustration, a sample rich in fatty acids could signal elevated concentrations of volatile elements linked with it [47]. Previous studies have also indicated that the primary production of flavor substances occurs through the oxidation of unsaturated fatty acids [48].

## 4. Conclusions

This study used GC-I-MS, GC-O-MS, and chemometrics to systematically elucidate the mechanisms underlying the divergence in volatile flavor profiles among four pork varieties: DLY, NX, RC, and DW. The main conclusions are as follows: Firstly, the volatile organic compounds (VOCs) were similar in type but significantly different in content among the four pork varieties. Most importantly, our PLSR and Pearson correlation analysis revealed a novel finding: tridecanal, a characteristic flavor compound unique to NX (only detected in NX), showed a strong positive correlation with the high vitamin E content and powerful total antioxidant capacity (T-AOC) in NX. This suggests that the unique fruity aroma of NX pork may not only originate from its fatty acid composition but also be closely related to its superior endogenous antioxidant system, which effectively modulates lipid oxidation pathways, promoting the formation and accumulation of specific flavor compounds like tridecanal. Furthermore, the characteristic cinnamon odor of RC was associated with compounds like (E)-2-decenal, and its high PUFA content was a prerequisite for its rich flavor. The fatty aroma of DW and DLY was closely related to aldehydes such as (E)-2-octenal. In conclusion, this study not only identified characteristic flavor markers that distinguish different pig breeds, but more importantly, it revealed for the first time a strong correlation between endogenous antioxidant factors (e.g., vitamin E) and the formation of characteristic flavor compounds in a specific breed (NX). This provides a new scientific perspective for understanding the formation mechanism of pork flavor from the antioxidant dimension and offers important reference value for the characterization and utilization of local pig genetic resources.

## Figures and Tables

**Table 1:** Key flavor compounds in different pork varieties and corresponding ROAV values.

Compounds	Threshold Value	Odor Perception	DLY	NX	RC	DW
(E)-2-Decenal	2.7	Waxy	1.31	0.81	0.90	0.92
Hexanoic acid	4.8	Fatty	1.02	0.50	0.50	0.52
1-Octanol	22	Fatty	1.09	1.27	1.17	1.02
(E,E)-2,4-Nonadienal	0.2	Strong, fatty, floral	3.44	1.51	5.98	0.00
(E)-2-Octenal	2.7	Nutty, fatty	3.39	2.70	3.62	3.05
3-phenyl-2-Propenal	0.081	Cinnamon odor	7.18	14.71	14.24	12.48
3-Octanone	1.3	herbaceous, Fruity warm odor	2.97	0.00	0.00	0.00
(Z,Z,Z)-9,12,15-Octadecatrien-1-ol	0.08	Floral	11.01	0.00	11.51	9.37
cis-4-Decenal	22	Fatty	20.33	0.00	0.00	18.32
Tridecanal	10	Fruity	0.00	30.38	0.00	0.00
1-Octen-3-ol	1	Mushroom	100.00	100.00	100.00	100.00

**Table 2:** Total fatty and fatty acid composition of pork varieties, unit: %. Peer lowercase letters are different, indicating a significant difference (*p* < 0.05).

Item	DLY	NX	RC	DW
IMF	1.42 ± 0.32 ^c^	3.17 ± 0.56 ^b^	3.72 ± 0.30 ^a^	3.15 ± 0.27 ^b^
C6:0	0.47 ± 0.05 ^b^	0.38 ± 0.07 ^b^	0.84 ± 0.13 ^a^	0.54 ± 0.07 ^b^
C8:0	ND	0.01 ± 0.01 ^ab^	0.01 ± 0.01 ^b^	0.02 ± 0.01 ^a^
C10:0	0.06 ± 0.01 ^b^	0.08 ± 0.01 ^ab^	0.05 ± 0.01 ^b^	0.09 ± 0.01 ^a^
C12:0	0.01 ± 0.01 ^b^	0.02 ± 0.01 ^a^	0.01 ± 0.01 ^b^	0.02 ± 0.01 ^a^
C13:0	0.09 ± 0.01 ^a^	0.06 ± 0.01 ^a^	0.09 ± 0.02 ^a^	0.08 ± 0.01 ^a^
C14:0	1.28 ± 0.09 ^ab^	1.47 ± 0.11 ^a^	1.09 ± 0.04 ^b^	1.35 ± 0.17 ^ab^
C15:0	0.03 ± 0.01 ^a^	0.02 ± 0.01 ^bc^	0.02 ± 0.01 ^ab^	0.02 ± 0.01 ^c^
C16:0	24.11 ± 0.83 ^b^	27.22 ± 0.30 ^a^	23.21 ± 0.37 ^b^	24.87 ± 0.57 ^b^
C17:0	0.12 ± 0.01 ^a^	0.11 ± 0.01 ^a^	0.12 ± 0.02 ^b^	0.08 ± 0.01 ^a^
C18:0	13.59 ± 0.50 ^b^	15.61 ± 0.68 ^a^	11.96 ± 0.22 ^c^	11.75 ± 0.29 ^c^
C20:0	0.09 ± 0.01 ^b^	0.06 ± 0.01 ^c^	0.18 ± 0.01 ^a^	0.06 ± 0.01 ^c^
C21:0	0.03 ± 0.01 ^b^	0.01 ± 0.01 ^b^	0.05 ± 0.01 ^a^	0.02 ± 0.01 ^b^
C22:0	0.41 ± 0.02 ^a^	0.30 ± 0.03 ^b^	0.46 ± 0.01 ^a^	0.28 ± 0.01 ^b^
C24:0	0.62 ± 0.06 ^b^	0.51 ± 0.04 ^b^	0.92 ± 0.07 ^a^	0.54 ± 0.03 ^b^
C15:1	1.10 ± 0.13 ^a^	0.79 ± 0.06 ^a^	1.06 ± 0.11 ^a^	1.00 ± 0.07 ^a^
C16:1	3.10 ± 0.25 ^ab^	2.86 ± 0.12 ^b^	3.01 ± 0.13 ^ab^	3.49 ± 0.18 ^a^
C17:1	0.39 ± 0.04 ^b^	0.35 ± 0.05 ^b^	0.62 ± 0.05 ^a^	0.39 ± 0.02 ^b^
C18:1n9t	0.15 ± 0. 01 ^a^	0.13 ± 0. 01 ^b^	0.16 ± 0. 01 ^a^	0.15 ± 0. 01 ^a^
C18:1n9c	38.98 ± 0.89 ^a^	36.37 ± 1.57 ^a^	37.30 ± 0.82 ^a^	39.68 ± 0.75 ^a^
C24:1	0.22 ± 0.01 ^ab^	0.17 ± 0.02 ^b^	0.27 ± 0.03 ^a^	0.17 ± 0.01 ^b^
C22:1n9	0.74 ± 0.07 ^b^	0.57 ± 0.05 ^b^	1.08 ± 0.09 ^a^	0.64 ± 0.04 ^b^
C18:2n6t	1.41 ± 0.10 ^b^	1.31 ± 0.06 ^b^	1.82 ± 0.15 ^a^	1.75 ± 0.10 ^a^
C18:2n6c	10.86 ± 0.79 ^b^	9.58 ± 0.85 ^b^	13.53 ± 0.90 ^a^	7.26 ± 0.37 ^c^
C18:3n3	0.89 ± 0.05 ^a^	0.96 ± 0.04 ^a^	0.59 ± 0.04 ^b^	0.89 ± 0.01 ^a^
C20:2	0.32 ± 0.01 ^a^	0.33 ± 0.02 ^a^	0.34 ± 0.02 ^a^	0.19 ± 0.01 ^b^
C20:3n6	0.31 ± 0.03 ^b^	0.26 ± 0.01 ^b^	0.46 ± 0.03 ^a^	0.27 ± 0. 01 ^b^
C20:4n6	0.04 ± 0.01 ^a^	0.02 ± 0.01 ^ab^	0.03 ± 0.01 ^a^	0.02 ± 0.01 ^b^
C22:2	0.34 ± 0.03 ^b^	0.27 ± 0.02 ^b^	0.50 ± 0.03 ^a^	0.29 ± 0. 01 ^b^
C22:6n3	0.20 ± 0.01 ^a^	0.11 ± 0.01 ^b^	0.25 ± 0.03 ^a^	0.09 ± 0.01 ^b^
SFA	40.96 ± 0.49 ^b^	45.92 ± 1.49 ^a^	39.02 ± 0.71 ^c^	39.73 ± 0.64 ^bc^
MUFA	44.67 ± 0.71 ^a^	41.24 ± 0.78 ^b^	43.48 ± 0.41 ^ab^	45.52 ± 0.44 ^a^
PUFA	14.39 ± 0.92 ^b^	12.84 ± 0.90 ^bc^	17.50 ± 1.05 ^a^	10.76 ± 0.40 ^c^
UFA	59.04 ± 0.71 ^ab^	54.08 ± 0.78 ^b^	60.98 ± 0.42 ^a^	56.28 ± 0.44 ^b^

**Table 3:** Analysis of antioxidant capacity characterization value and endogenous antioxidant factors. Peer lowercase letters are different, indicating a significant difference (*p* < 0.05).

Item	DLY	NX	RC	DW
T-AOC/ABTS (mM Trolox)	0.27 ± 0.01 ^b^	0.33 ± 0.01 ^a^	0.28 ± 0.05 ^b^	0.29 ± 0.02 ^b^
T-AOC/FRAP (mmol/L FeSO_4_)	0.19 ± 0.01 ^c^	0.33 ± 0.01 ^a^	0.25 ± 0.02 ^b^	0.22 ± 0.02 ^b^
DPPH (mM Trolox)	1109.30 ± 0.01 ^c^	1791.22 ± 0.08 ^a^	1549.18 ± 0.01 ^b^	1063.06 ± 0.01 ^c^
OH•− (U/mg prot)	26.02 ± 1.38 ^b^	29.10 ± 1.10 ^b^	29.14 ± 2.21 ^b^	38.20 ± 3.19 ^a^
SOD (U/mg prot)	24.66 ± 0.95 ^b^	53.06 ± 3.94 ^a^	44.22 ± 4.12 ^a^	50.90 ± 2.94 ^a^
CAT (U/mg prot)	6.04 ± 1.18 ^b^	10.39 ± 0.90 ^b^	8.03 ± 1.07 ^b^	17.07 ± 1.88 ^a^
GSH-Px (U/mg prot)	3.11 ± 0.51 ^b^	6.56 ± 0.53 ^a^	5.12 ± 0.52 ^ab^	3.13 ± 0.96 ^b^
TPC (mg GAE/g)	0.73 ± 0.02 ^a^	0.69 ± 0.02 ^ab^	0.65 ± 0.02 ^b^	0.71 ± 0.02 ^ab^
Vitamin C (μg/g)	21.80 ± 1.34 ^b^	28.37 ± 2.34 ^a^	23.53 ± 1.11 ^b^	30.63 ± 1.49 ^a^
Vitamin E (μg/g)	14.43 ± 0.74 ^b^	71.28 ± 1.36 ^a^	5.21 ± 0.25 ^c^	5.87 ± 0.80 ^c^

## Data Availability

The original contributions presented in this study are included in the article/Supplementary Materials. Further inquiries can be directed to the corresponding author.

## Supplementary Materials

The following supporting information can be downloaded at: https://www.mdpi.com/article/10.3390/foods14203580/s1, Table S1: GC-IMS comparison of volatile compounds in different pork varieties. Table S2. The relative contents of VOCs in different breed pork were determined by SPME-GC-O-MS.
