# Supplementary material for: Exploring the Relationship of Antioxidant Characteristics and Fatty Acids with Volatile Flavor Compounds (VOCs) by GC-IMS and GC-O-MS in Different Breeds of Pigs
Source: Foods. 2025 Oct 21;14(20):3580. doi: 10.3390/foods14203580 (PMC12563706; doi:10.3390/foods14203580)
Supplement: Supplementary file 1 [file foods-14-03580-s001.zip › foods-3837015-supplementary.pdf]

Table S1 GC-IMS comparison of volatile compounds in different pork varieties

Compounds	CAS#	RI	Rt[sec]	Intensity(V)			
				DLY	NX	RC	DW
Aldehydes							
3-methylbutanal	C590863	661.20	164.07	247.44±38.28[c]	425.46±60.90[b]	303.96±38.28[bc]	588.25±59.29[a]
butanal	C123728	586.00	127.81	415.83±49.27[b]	550.37±54.24[b]	481.39±29.87[b]	735.28±44.32[a]
Heptanal(M)	C111717	872.30	367.11	717.67±34.74[a]	270.07±39.03[c]	721.43±19.96[a]	518.19±56.51[b]
(E)-2-octenal	C2548870	1061.90	710.48	543.41±15.54[b]	232.91±39.09[c]	796.91±47.50[a]	589.6±19.47[b]
Nonanal	C124196	1103.10	791.13	713.94±42.38[a]	271.1±36.24[c]	495.46±21.36[b]	348.29±54.19[c]
2-Hexenal	C505577	845.00	334.05	444.62±19.06[a]	335.46±41.08[a]	425.34±58.65[a]	316.35±40.50[a]
phenylacetaldehyde	C122781	1037.80	663.41	760.76±18.24[a]	453.19±46.46[b]	242.55±23.43[c]	827.19±31.53[a]
hexanal	C66251	810.00	291.57	175.92±47.06[b]	115.99±27.87[b]	421.32±60.54[a]	129.22±20.81[b]
Heptanal(D)	C111717	898.70	403.23	356.59±52.29[b]	691.71±37.16[a]	296.51±21.58[b]	338.91±27.56[b]
pentanal	C110623	698.60	185.98	561.48±40.54[b]	492.65±62.08[b]	519.4±34.21[b]	805.56±33.16[a]
Propanal	C123386	746.60	228.03	817.74±26.27[a]	303.63±34.82[d]	619.23±22.49[b]	415.6±50.32[c]
2-Butenal,3-methyl-	C107868	639.00	299.57	204.25±25.42[b]	368.58±77.39[a]	192.11±27.58[b]	288.98±46.04[ab]
Alcohols							
2-Furanmethanol,5-methyl-	C3857258	955.20	506.82	475.98±47.23[b]	190.63±26.63[c]	609.42±42.24[a]	506.41±40.47[ab]
1-Propanethiol	C107039	620.70	144.55	653.82±44.19[a]	659.11±38.21[a]	351.1±42.78[b]	303.85±55.85[b]
2-octanol	C123966	1008.60	606.27	813.69±27.30[a]	530.22±43.53[c]	700.3±24.89[ab]	631.21±64.55[b]
n-Hexanol	C111273	885.00	382.56	942.2±46.78[a]	883.75±45.99[b]	740.43±30.05[c]	735.52±42.86[c]
1-pentanol(D)	C71410	774.60	252.03	884.37±15.88[a]	494.17±55.06[c]	720.76±38.5[b]	721.78±51.55[b]
Octan-3-ol	C589980	986.30	563.87	1131.02±30.79[b]	1158.3±11.47[b]	1304.69±64.57[a]	1104.31±11.05[b]
Propanol(M)	C71238	544.60	107.86	2239.31±58.02[b]	2216.13±52.62[b]	2639.96±55.64[a]	2243.34±57.67[b]

Propanol(D)	C71238	604.70	136.80	870.2±18.76[a]	202.55±28.10[d]	353.14±23.59[c]	616.76±35.68[b]
1-pentanol(M)	C71410	776.60	253.73	437.02±17.53[b]	405.76±48.29[b]	535.09±40.60[ab]	606.39±60.45[a]
3-pentanol	C584021	708.40	194.45	608.51±35.5[b]	398.84±40.68[c]	715.77±40.85[ab]	759.77±40.87[a]
Linalooloxide(pyranoid)	C60047178	710.00	385.43	550.71±12.96[b]	551.98±35.12[b]	551.67±48.68[b]	781.25±25.28[a]
				Esters			
2-Furancarboxylicacid,methylester(M)	C611132	970.20	534.37	558.78±14.9[b]	664.06±50.37[a]	642.14±18.1[a]	558.92±9.52[b]
2-Furancarboxylicacid,methylester(D)	C611132	969.00	532.18	523.67±40.69[b]	208.74±29.77[c]	701.51±38.76[a]	596.65±48.53[ab]
Sarin	C107448	807.80	288.95	518.24±22.54[b]	745.85±76.38[a]	553.07±30.27[b]	315.09±41.02[c]
Ethylformate	C109944	630.40	149.21	547.5±50.88[a]	655.15±45.29[a]	128.09±25.24[c]	365±39.41[b]
Methylacetate	C79209	548.70	109.83	654.81±41.61[a]	579.92±26.65[a]	202.5±36.65[c]	420.21±51.16[b]
n-Propylacetate	C109604	708.70	194.75	703.03±38.39[a]	265.22±27.88[c]	466.71±24.33[b]	299.81±43.02[c]
ethyl2-methylpropanoate	C97621	781.60	258.11	413.21±14.94[b]	390.56±39.98[b]	498.33±30.24[ab]	568.91±52.69[a]
Aceticacidbutylester	C123864	814.80	297.46	465.19±30.33[b]	687.41±47.67[a]	509.49±23.45[b]	219.41±34.87[c]
Ethylbutyrate	C105544	796.10	274.74	655.88±19.62[a]	618.35±44.50[a]	433.66±37.04[b]	722.22±38.67[a]
Hexylacetate	C142927	1011.00	610.94	445.06±46.94[a]	210.17±17.38[c]	312.09±26.46[b]	131.94±17.66[c]
Butylbutanoate	C109217	651.00	135.58	360.06±22.79[a]	290.09±62.64[a]	165.47±18.09[b]	398.8±44.99[a]
				Ketone			
Methylisobutylketone	C108101	742.70	224.32	433.43±8.42[b]	622.59±51.75[a]	405.28±6.84[b]	553.41±6.27[ab]
2-pentanone	C107879	688.10	177.02	497.15±26.76[a]	247.15±47.05[b]	145.65±22.90[c]	515.77±32.9[a]
2,3-butanedione	C431038	596.10	132.70	197.93±12.01[b]	178.87±14.27[b]	140.52±24.12[b]	427.12±57.97[a]
2,3-pentanedione(M)	C600146	610.40	139.58	503.73±27.99[a]	486.11±25.41[a]	330.28±51.53[b]	223.92±37.00[c]
2-heptanone	C110430	888.20	386.43	1118.72±53.61[a]	1137.24±51.04[a]	1113.58±54.87[a]	1169.96±49.63[a]
2,3-Pentanedione(D)	C600146	691.00	179.36	2127.78±45.11[b]	2191.4±18.91[b]	2286.2±60.87[a]	2191.94±13.73[b]
				Acids			

2-Methylpropionicacid	C79312	789.00	266.13	781.37±28.95[a]	360.46±39.93[c]	603.16±21.15[a]	415.71±61.13[b]
Propanoicacid	C79094	662.20	164.53	1136.16±15.51[ab]	1271.88±11.41[a]	1197±53.28[ab]	1008.82±10.91[b]
Furans							
2-pentylfuran	C3777693	992.20	574.62	741.54±18.63[b]	713.64±51.36[b]	935.3±7.76[a]	875.77±24.8[a]
2-Ethylfuran	C3208160	683.90	174.99	515.15±20.65[a]	661.62±28.41[a]	729.08±59.72[a]	309.52±38.88[c]
tetrahydrofuran	C109999	609.00	138.91	1272.21±23.19[b]	1226.05±5.69[b]	1318.56±66.93[a]	1252.76±6.57[b]
Hydrocarbon							
?alpha?-Phellandrene	C99832	1007.10	603.40	1441.27±18.19[a]	1202.28±47.67[a]	1414.29±10.69[a]	1193.15±21.32[c]
1-Octen-3-ol	C3391864	992.50	575.18	660.57±40.54[a]	184.62±29.48[c]	640.24±25.66[a]	435.3±39.87[b]
1,2-Dimethoxyethane	C110714	632.20	150.06	274.95±15.25[b]	372.77±17.64[a]	289.54±43.71[b]	159.48±21.34[c]
Heptane,2,2,4,6,6-pentamethyl-	C13475826	997.20	583.86	719.13±34.74[a]	234.21±33.82[c]	761.02±31.54[a]	534.03±43.11[b]
ethers							
Diethyleneglycoldimethylether	C111966	953.10	503.01	498.2±48.46[b]	398.98±43.59[b]	462.27±27.25[b]	732.26±35.99[a]

DLY (Duroc×Landrace×Yorkshire pig); NX (Yorkshire×Ningxiang pig); RC (Rongchang pig); DW (Duroc×Wujin pig). The values are presented by means ± standard deviations. a, b mean values with various superscripts in a row were significant differences ($p < 0.05$). n = 6.

Table S2. The relative contents of VOCs in different breed pork were determined by SPME-GC-O-MS.

Compounds	CAS	Order perception	Experimental LRI	Referenced LRI	Experimental LRI	Referenced LRI	Relative content %（DB-WAX）				Relative content %（DB-5）			
							DLY	NX	RC	DW	DLY	NX	RC	DW
							Aldehydes							
(E,E)-2,4-Decadienal	25152 - 84 - 5	fatty	1812	1806	1812	1312	0.12±0.01^{b}	0.14±0.02^{b}	0.14±0.02^{b}	0.31±0.01^{a}	0.10±0.01^{b}	0.22±0.01^{a}	0.22±0.02^{a}	0.18±0.02^{a}
(E,E)-2,4-Nonadienal	5910-87-2	fruity	1772	1697	1772	-	0.10±0.02^{a}	0.11±0.02^{a}	0.10±0.0^{a}	-	0.04±0.32^{a}	-	0.06±0.01^{a}	-
(E)-2-Decenal	3913 - 81 - 3	waxy	2496	1641	2496	1266	0.36±0.03^{a}	0.22±0.02^{b}	0.22±0.02^{b}	0.23±0.01^{b}	0.39±0.03^{b}	0.36±0.01^{b}	0.58±0.02^{a}	0.45±0.02^{b}
(E)-2-Dodecenal	20407 - 84 - 5	fatty	2261	1748	2261	1363	0.16±0.03^{a}	0.09±0.02^{b}	0.10±0.01^{b}	0.13±0.01^{a}	0.12±0.03^{b}	0.30±0.01^{a}	0.28±0.01^{a}	0.18±0.02^{b}
(E)-2-Hexenal	6728 - 26 - 3	strong fruity, vegetable-like	1629	1219	1629	850	0.10±0.02^{a}	0.04±0.01^{b}	0.06±0.01^{b}	-	-	-	-	-
2-ethyl-2-Hexenal	645 - 62 - 5		1618	1327	1618	1002	0.26±0.03^{a}	0.24±0.02^{a}	0.23±0.03^{a}	0.12±0.01^{b}	0.36±0.03^{b}	0.66±0.01^{a}	-	-
(E)-2-Nonenal	18829-56-6	fatty	1802	1532	1802	1154	-	-	0.43±0.01	-	0.23±0.01^{b}	0.29±0.01^{b}	0.45±0.01^{a}	0.35±0.01^{b}
3-phenyl-2-Propenal	104 - 55 - 2	cinnamon odor	1423	2003	1423	1260	0.08±0.01^{b}	-	0.13±0.01^{a}	0.09±0.01^{b}	-	-	-	-

Benzaldehyde	100 - 52 - 7	fruity, sweet	1244	1518	1244	1346	4.53±0.57^{a}	3.76±0.39^{b}	4.10±0.14^{a}	4.38±0.32^{a}	4.39±0.57^{a}	4.18±0.32^{a}	-	-
4-ethyl-Benzaldehyde	4748 - 78 - 1		1644	1703	1644	-	0.11±0.02^{a}	0.15±0.02^{a}	0.09±0.01^{a}	-	-	-	-	-
Decanal	112 - 31 - 2	odor of citrus	1461	1495	1961	-	0.26±0.04	-	-	-	0.18±0.04^{b}	0.31±0.07^{a}	-	-
Dodecanal	112 - 54 - 9	soap	2954	-	1454	1406	-	2.99±0.02	-	-	0.14±0.01^{b}	0.21±0.01^{b}	0.14±0.01^{b}	0.81±0.02^{a}
Heptanal	111 - 71 - 7	fatty	1159	1173	1859	-	3.94±0.42^{a}	3.47±0.19^{a}	3.43±0.24^{a}	3.33±0.15^{a}	2.36±0.34^{a}	1.16±0.13^{b}	1.78±0.24^{ab}	1.79±0.21^{ab}
Hexadecanal	629 - 80 - 1	burnt	1265	2131	1265	1816	0.42±0.04^{b}	1.03±0.11^{a}	0.41±0.01^{b}	0.06±0.02^{c}	-	-	-	-
Hexanal	66 - 25 - 1	grassy, green	1119	1069	1119	-	35.59±2.93^{a}	29.69±2.04^{b}	34.71±1.26^{a}	33.26±1.22^{ab}	43.12±3.03^{a}	35.11±2.22^{b}	38.75±2.26^{b}	41.46±2.84^{a}
Nonanal	124 - 19 - 6	citrus, fatty	-	1387	1101	-	7.16±0.87^{b}	9.33±0.60^{a}	7.38±0.65^{b}	7.04±0.18^{b}	8.93±1.87^{b}	10.19±1.67^{ab}	10.34±1.55^{ab}	14.86±2.60^{a}
Octanal	124 - 13 - 0	green, citrus, lemon	1233	1280	1233	1005	4.17±0.41^{a}	3.26±0.13^{b}	3.41±0.17^{b}	3.34±0.07^{b}	3.86±0.41^{a}	3.73±0.42^{a}	3.66±0.21^{a}	3.85±0.34^{a}
Pentadecanal-	2765 - 11 - 9	waxy	2126	2034	1626	1715	0.40±0.02^{b}	0.56±0.06^{b}	0.91±0.09^{a}	1.10±0.13^{a}	0.12±0.01^{b}	0.25±0.01^{a}	-	0.03±0.01^{c}
Pentanal	110 - 62 - 3	green	1484	-	1484	-	3.86±0.25^{ab}	3.14±0.23^{b}	4.20±0.16^{a}	4.03±0.20^{a}	-	-	-	-
Tetradecanal	124 - 25 - 4	fatty	1794	1914	1794	1606	0.28±0.03^{b}	0.40±0.02^{ab}	0.58±0.03^{a}	0.61±0.08^{a}	0.40±0.02^{b}	0.63±0.03^{a}	0.62±0.03^{a}	0.50±0.02^{b}

Undecanal	112 - 44 - 7	sweet	1557	1598	1557	-	0.10±0.02^{a}	0.05±0.02^{a}	-	-	-	-	-	-
(E)-2-Heptenal,	18829 - 55 - 5	Pungent green	1883	-	983	947	-	-	-	-	1.15±0.22^{b}	-	2.49±0.02^{a}	1.21±0.02^{b}
Tridecanal	10486 - 19 - 8	fruity	1910	1824	1910	1518	-	-	-	-	-	0.09±0.01^{a}	-	-
(E)-2-Octenal	2548 - 87 - 0	nut, fat	1207	1215	1207	-	1.91±0.06^{a}	1.71±0.04^{b}	1.87±0.02^{a}	1.77±0.04^{b}	0.80±0.06^{b}	1.21±0.04^{a}	1.80±0.02^{a}	1.42±0.04^{a}
cis-4-Decenal	21662 - 09 - 9	fatty	1545	1537	1545	-	-	-	-	-	0.57±0.12^{a}		-	0.18±0.01^{b}
(E)-2-Undecena	53448-07-0	resin, soap	1432	1369	1732	1722	-	0.14±0.02	-	-	-	-	-	-
							Alcohols							
1,4-Butanediol	110 - 63 - 4		1145	1134	1145	-	0.06±0.02^{c}	0.28±0.02^{a}	0.14±0.02^{b}	0.15±0.02^{b}	-	-	-	-
1-Butanol	71 - 36 - 3	fatty	1455	1447	1455	-	0.04±0.02^{b}	0.11±0.02^{a}	0.02±0.01^{b}	-	-	-	-	-
1-Dodecanol	112 - 53 - 8	alcohol-like odor	1852	1970	1352	1474	-	0.26±0.03^{a}	0.22±0.02^{a}	0.18±0.01^{b}	-	0.18	-	-
1-Heptanol	111 - 70 - 6	floral	1490	1459	1490	967	1.85±0.10^{a}	1.28±0.09^{b}	1.30±0.05^{b}	1.26±0.05^{b}	0.66±0.10^{b}	0.89±0.05^{a}	-	0.60±0.09^{b}

1-Hexadecanol	36653 - 82 - 4		1740	1430	1740	1881	-	-	0.05±0.02^{a}	0.02±0.01^{a}	-	-	-	-
1-Hexanol	111 - 27 - 3	sweet, green	1251	1252	1251	862	0.98±0.10^{b}	1.37±0.14^{a}	0.93±0.03^{b}	0.90±0.04^{b}	0.34±0.10^{b}	0.60 ±0.04^{a}	0.61±0.03^{a}	0.47±0.14^{b}
2-ethyl-1-Hexanol	104 - 76 - 7	aromatic	1469	1492	1469	-	0.21±0.02^{b}	0.61±0.06^{a}	0.55±0.06^{a}	0.31±0.05^{b}	0.12±0.02^{a}	0.22±0.05^{a}	-	-
1-Nonanol	143 - 08 - 8	floral	1477	1663	1477	-	0.16±0.04^{b}	0.65±0.11^{a}	0.22±0.02^{b}	-	-	-	-	-
1-Pentanol	71 - 41 - 0	mild odor	1214	1256	1214	-	1.85±0.21^{a}	1.75±0.16^{a}	1.86±0.07^{a}	1.79±0.10^{a}	1.53±0.21^{b}	2.08±0.10^{ab}	3.59±0.07^{a}	3.54±0.16^{a}
1-Tetradecanol	112 - 72 - 1		1252	1246	1252	-	0.06±0.02^{a}	0.02±0.01^{b}	0.06±0.01^{a}	0.05±0.01^{a}	-	-	-	-
2,3-dimethyl-2,3-Butanediol	76-09-5		1369	1338	1969	-	0.07±0.02^{b}	0.09±0.01^{b}	0.16±0.03^{a}	-	0.93±0.01	-	-	-
(Z,Z,Z)-9,12,15-Octadecatrien-1-ol	506 - 44 - 5	floral	1293	-	1293	-	1.09±0.02^{a}	-	1.09±0.00^{a}	1.09±0.01^{a}	-	-	-	-
n-Pentadecanol	629 - 76 - 5	faint odor of alcohol	2457	1256	2457	-	0.03±0.01^{b}	0.05±0.01^{ab}	0.07±0.02^{a}	0.06±0.01^{a}	-	-	-	-
n-Tridecan-1-ol	112-70-9	pleasant odor	2119	2063	1219	1572	-	0.06±0.01^{b}	0.12±0.02^{a}	0.07±0.02^{b}	-	-	-	-
1-Hepten-4-ol	3521-91-3		1561	1546	1561	-	-	0.12±0.02^{a}	0.07±0.02^{a}	0.08±0.02^{a}	-	-	-	-
Linalool	78 -	floral	1507	1549	2307	-	0.07±	0.16±	-	0.06±0.	-	-	-	-

	70 - 6						0.02^{b}	0.02^{a}		02^{b}				
Cyclooctyl alcohol	696 - 71 - 9		1745	1622	1745	-	0.42±0.03^{b}	0.40±0.02^{b}	0.59±0.03^{a}	-	-	-	-	-
3-methyl-3-Buten-1-ol	763 - 32 - 6	sweet	1699	1252	1699	-	0.08±0.01^{a}	-	-	0.09±0.02^{a}	-	-	-	-
1-Octanol	111 - 87 - 5	fatty	1848	1561	1848	1272	2.41±0.22^{a}	2.82±0.22^{a}	2.22±0.10^{a}	2.11±0.10^{a}	1.66±0.22^{b}	2.53±0.21^{a}	2.29±0.10^{a}	1.65±0.20^{b}
1-Octen-3-ol	3391 - 86 - 4	mushroom	1449	1453	1449	-	7.63±1.45^{b}	9.74±0.68^{a}	8.93±0.73^{a}	9.43±0.37^{a}	10.63±0.75^{b}	16.48±1.37^{a}	8.76±0.73^{b}	11.53±0.68^{b}
(Z)-3,7-dimethyl-2,6-Octadien-1-ol	106 - 25 - 2		1324	1301	1324	-	0.05±0.01^{a}	-	-	0.06±0.02^{a}	-	-	-	-
2-Nonen-1-ol	22104 - 79 - 6		1246	1239	1246	-	3.27±1.34	-	-	-	3.20±0.89^{a}	-	2.77±0.93^{a}	-
(E)-2-Octen-1-ol,	18409 - 17 - 1	Clear colourless liquid; Meaty, roasted aroma	1731	1618	1731	1066	1.29±0.05^{a}	1.35±0.10^{a}	1.43±0.07^{a}	1.06±0.09^{b}	2.12±0.05^{a}	1.36±0.09^{b}	2.12±0.07^{a}	1.09±0.10^{b}
							Ketones							
1-Hepten-3-one	2918 - 13 - 0		1651	1295	1651	-	0.11±0.02^{a}	0.07±0.01^{b}	0.09±0.02^{ab}	0.05±0.01^{b}	0.04±0.02^{a}	-	0.06±0.02^{a}	-
2,3-Octanedione	585 -	fatty	1472	1319	1472	982	4.21±	4.20±	4.62±0.2	4.52±0.	5.13±0.	8.83±0.19^{a}	8.27±0.23^{a}	7.68±0.36

	25 - 1						0.32^{b}	0.36^{b}	3^{a}	19^{a}	32^{c}			^{b}
2-Heptanone	110 - 43 - 0	fruity	1620	1184	1620	1689	-	-	-	0.20±0.02	0.72±0.03^{a}	0.88±0.02^{a}	0.36±0.01^{b}	0.55±0.02^{b}
6-methyl-2-Heptanone	928 - 68 - 7		2065	1229	2065	-	0.14±0.02^{a}	-	0.11±0.01^{a}	0.06±0.02^{b}	-	-	-	-
2-Octanone	111 - 13 - 7	earthy	1625	1276	1625	-	0.06±0.02^{a}	0.08±0.02^{a}	-	-	-	-	-	-
2-Pentadecanone	2345 - 28 - 0		2126	2021	1326	1698	0.03±0.01^{a}	0.03±0.01^{a}	0.02±0.01k^{a}	-	-	-	-	-
3-Heptanone	106 - 35 - 4		1489	1164	1489	889	0.06±0.01^{b}	0.09±0.02^{a}	0.05±0.02^{b}	0.05±0.01^{b}	-	-	-	-
3-Nonen-2-one	14309 - 57 - 0	fatty	1064	-	1064	1336	-	-	0.21±0.02^{a}	0.19±0.01^{a}	0.11±0.01^{b}	0.17±0.01^{a}	0.19±0.02^{a}	0.05±0.01^{b}
3-Octanone	106 - 68 - 3	fruity	1279	1240	1279	970	0.35±0.06	-	-	-	-	-	-	-
3-Octen-2-one	1669 - 44 - 9		-	1403	-	-	0.21±0.04^{a}	0.16±0.02^{b}	0.09±0.01^{c}	0.16±0.02^{b}	-	-	-	-
6,10-dimethyl-5,9-Undecadien-2-one	3796-70-1	floral	1824	1862	1824	1455	-	-	0.05±0.02	-	2.74±0.35^{b}	-	3.35±0.66^{a}	-
6-methyl-5-Hepten-2-one,	110 - 93 - 0	fruity	1331	1332	1531	-	0.14±0.02^{b}	0.24±0.01^{a}	0.12±0.01^{b}	0.15±0.02^{b}	-	-	-	-
							Acid							
(Z,Z,Z)-9,12,15-Octadecatrienoic acid	463 - 40 - 1	fatty	3319	3292	1319	2116	-	-	1.32±0.01	-	-	-	-	-

Acetic acid	64 - 19 - 7		1328	1429	1228	625	-	-	1.37±0.02	-	-	-	-	-
Dodecanoic acid	143 - 07 - 7	slight odor of bay oil	2710	2509	2710	-	0.16±0.02^{ab}	0.26±0.08^{a}	0.09±0.02^{b}	-	-	-	-	-
Hexanoic acid	142 - 62 - 1	fatty	1906	1853	1106	-	0.57±0.06^{a}	0.25±0.02^{b}	0.21±0.03^{b}	0.24±0.01^{b}	-	-	-	-
n-Decanoic acid	334 - 48 - 5	fatty, rancid	1476	1495	2076	-	0.27±0.04^{b}	0.47±0.09^{a}	0.31±0.04^{b}	0.29±0.04^{b}	-	-	-	-
n-Hexadecanoic acid	57 - 10 - 3	waxy	1982	2910	982	972	0.87±0.15^{a}	0.90±0.04^{a}	-	-	-	-	-	-
Nonanoic acid	112 - 05 - 0	coconut aroma	2052	2178	1952	-	0.19±0.03^{ab}	0.23±0.03^{a}	0.12±0.01^{b}	0.14±0.01^{b}	-	-	-	-
Tetradecanoic acid	544 - 63 - 8	waxy	2579	2672	1179	1765	0.15±0.02^{b}	0.33±0.05^{a}	-	0.14±0.02^{b}	-	-	-	-
							Esters							
(Z)-2,6-Octadien-1-ol, 3,7-dimethyl-, acetate	141-12-8		1965	1699	1965	1363	-	-	-	1.68±0.01	-	-	-	-
Acetic acid, butyl ester	123 - 86 - 4	pungent, sweet	1077	1049	1277	802	0.07±0.01^{a}	0.10±0.01^{a}	0.05±0.02^{b}	0.08±0.01^{a}	-	-	-	-
Acetic acid, hexyl ester	142 - 92 - 7	fruity	1216	1261	1077	1011	-	0.07±0.02	-	-	-	-	-	-
Allyl 2-ethyl butyrate	7493 - 69 - 8	oily fruity	1653	1636	1153	-	0.49±0.06^{b}	0.80±0.06^{a}	0.39±0.07^{b}	0.40±0.02^{b}	-	-	-	-

		odour												
Butanoic acid, octyl ester	110 - 39 - 4	fruity	1630	1602	1330	1372	-	-	0.12±0.00	-	-	-	-	-
Propanoic acid, 2-hydroxy-2-methyl-, ethyl ester	80 - 55 - 7		1580	1481	1080	-	-	0.06±0.01^{a}	-	0.05±0.01^{a}	-	-	-	-
Propanoic acid, 2-methyl-, 2-ethyl-3-hydroxyhexyl ester	74367 - 31 - 0		1127	-	1127	1373	-	0.13±0.01^{a}	-	0.16±0.01^{a}	-	-	-	-
							Hydrocarbons							
Dodecane	112 - 40 - 3		1175	1182	1275	1201	0.36±0.10^{b}	0.38±0.02^{b}	0.64±0.08^{a}	0.56±0.07^{ab}	0.14±0.10^{b}	0.26±0.02^{b}	2.06±0.08^{a}	1.48±0.06^{a}
Hexadecane	544 - 76 - 3		1617	1597	1017	1601	0.17±0.03^{b}	0.77±0.21^{a}	-	-	0.13±0.03^{a}	-	-	0.15±0.21^{a}
n-Hexane	110 - 54 - 3	gasoline-like odor	1981	-	981	-	0.30±0.06^{a}	0.26±0.07^{b}	0.27±0.10^{ab}	0.10±0.02^{c}	-	-	-	-
Octadecane	593 - 45 - 3		1678	1796	1678	-	0.18±0.02^{ab}	0.24±0.03^{a}	0.19±0.01^{ab}	0.16±0.02^{b}	0.08±0.01^{b}	0.09±0.02^{b}	0.05±0.01^{b}	0.33±0.03^{a}
Tetradecane	629 - 59 - 4		1883	-	1283	1391	0.52±0.14^{ab}	0.45±0.12^{b}	0.61±0.02^{a}	0.55±0.05^{ab}	0.11±0.01^{b}	0.13±0.04^{b}	0.23±0.02^{a}	0.21±0.01^{a}
Tridecane	629 - 50 - 5		1310	1287	1410	1301	0.25±0.04^{b}	0.43±0.05^{a}	0.55±0.05^{a}	0.46±0.07^{a}	0.18±0.02^{b}	0.28±0.04^{b}	0.62±0.02^{a}	0.50±0.03^{a}
1-(1-methylethyl)-C	1462 -		1500	1410	2500	-	-	-	0.65±0.0	-	-	-	-	-

yclopentene	07 - 3								2^{a}					
D-Limonene	5989 - 27 - 5	citrus, fragant	1182	1180	2282	-	0.19±0.04^{b}	0.30±0.02^{a}	-	-	-	-	-	-
3-Nonyne	20184 - 89 - 8		1087	1021	1387	-	0.61±0.05^{b}	0.43±0.03^{b}	1.04±0.03^{a}	0.56±0.04^{b}	0.54±0.05^{b}	0.90±0.04^{a}	1.05±0.03^{a}	0.91±0.03^{a}
Heterocyclic compound														
2-pentyl-Furan	3777 - 69 - 3	2-pentylfuran	1129	1218	1089	988	0.52±0.16^{b}	0.69±0.03^{a}	0.63±0.05^{a}	-	0.91±0.06^{b}	1.30±0.08^{ab}	1.93±0.15^{a}	1.88±0.13^{a}
o-Xylene	95-47-6	aromatic odor	0	1175	-	889	-	0.08±0.02^{a}	-	0.08±0.01^{a}	-	-	-	-
Toluene	108 - 88 - 3	sweet	1145	1021	1245	-	1.19±0.28^{b}	2.19±0.19^{b}	1.20±0.10^{b}	7.33±2.61^{a}	0.46±0.12^{b}	3.50±1.55^{a}	0.40±0.10^{b}	0.21±0.09^{b}
Ethylbenzene	100-41-4		1092	1106	1992	-	-	-	-	0.07±0.01^{a}	-	-	-	-
1,3-dimethyl-Benzene	108-38-3		1401	1115	1401	-	-	0.08±0.01^{a}	-	0.08±0.01^{a}	0.10±0.01^{a}	-	0.12±0.01^{a}	-
Benzothiazole	95 - 16 - 9		1931	1947	1331	1226	-	0.09±0.02^{a}	0.04±0.01^{b}	0.05±0.02^{b}	-	-	-	-
							1.70	3.12	1.86	7.61	1.48	4.79	2.45	2.10
2,4-Di-tert-butylphenol	96 - 76 - 4		1549	1552	1549	-	0.05±0.02^{b}	0.06±0.02^{b}	0.12±0.03^{a}	0.06±0.02^{b}	-	-	-	-
p-Cresol	106 - 44 - 5	dry tarry, medici	2044	2078	1070	1084	0.10±0.02^{a}	0.06±0.01^{a}	-	-	-	-	-	-

		ne												
m-tert-butyl-Phenol	585-34-2		2208	2287	2208	1293	-	-	-	-	0.12±0.01^{b}	0.26±0.04^{a}	-	0.10±0.01^{b}
Phenols														
2-(2-ethoxyethoxy)-Ethanol	111-90-0	fruity	1671	1619	1071	1007	0.16±0.02^{b}	0.38±0.04^{a}	0.31±0.01^{ab}	0.27±0.02^{b}	-	-	-	-
2-ethoxy-Ethanol	110-80-5	sweet, pleasant	1252	1239	752		-	-	0.06±0.01	-	-	-	-	-
Nitrogen-containing compounds														
Acetonitrile	75-05-8	fatty	1139	1026	1139	-	3.97±0.35^{b}	6.08±0.46^{a}	3.87±0.32^{b}	5.04±0.33^{a}	-	-	-	-
	95-47-6	aromatic odor	0	1175	-	889	-	0.08±0.02^{a}	-	0.08±0.01^{a}	-	-	-	-
	108-88-3	sweet	1145	1021	1245	-	1.19±0.28^{b}	2.19±0.19^{b}	1.20±0.10^{b}	7.33±2.61^{a}	0.46±0.12^{b}	3.50±1.55^{a}	0.40±0.10^{b}	0.21±0.09^{b}
